# Human T-Cell Lymphotropic Virus Types 1 and 2 Seropositivity among Blood Donors at Mbarara Regional Blood Bank, South Western Uganda

**DOI:** 10.1155/2016/1675326

**Published:** 2016-02-29

**Authors:** Patience Uchenna Tweteise, Bernard Natukunda, Joel Bazira

**Affiliations:** ^1^Department of Microbiology, Faculty of Medicine, Mbarara University Science & Technology, P.O. Box 1410, Mbarara, Uganda; ^2^Department of Medical Laboratory Science, Faculty of Medicine, Mbarara University of Science & Technology, P.O. Box 1410, Mbarara, Uganda

## Abstract

*Background. *The human T-cell lymphotropic virus types 1 and 2 (HTLV 1/2) are retroviruses associated with different pathologies. HTLV-1 causes adult T-cell leukemia/lymphoma (ATL) and HTLV-1-associated myelopathy/tropical spastic paraparesis (HAM/TSP); HTLV-2 is not clearly associated with a known clinical disease. Both viruses may be transmitted by whole blood transfusion, from mother to child predominantly through breastfeeding, and by sexual contact. Presently, none of the regional blood banks in Uganda perform routine pretransfusion screening for HTLV. The aim of this study was to determine the prevalence of anti-human T-cell lymphotropic virus types 1/2 (HTLV-1/2) antibodies among blood donors at Mbarara Regional Blood Bank in South Western Uganda. A cross-sectional study was conducted between June 2014 and September 2014.* Methodology*. Consecutive blood samples of 368 blood donors were screened for anti-HTLV-1/2 antibodies using an enzyme linked immunosorbent assay (ELISA). Samples reactive on a first HTLV-1/2 ELISA were further retested in duplicate using the same ELISA. Of the three hundred and sixty-eight blood donors (229 (62.2%) males and 139 (37.8%) females), only two male donors aged 20 and 21 years were HTLV-1/2 seropositive, representing a prevalence of 0.54%.* Conclusion*. HTLV-1/2 prevalence is low among blood donors at Mbarara Regional Blood Bank. Studies among other categories of people at risk for HTLV 1/2 infection should be carried out.

## 1. Introduction

Almost 10–20 million people in the world are thought to be infected by human deltaretroviruses, namely, human T-cell lymphotropic virus (HTLV) types 1 and 2 [1].

Human T-cell lymphotropic virus type 1 (HTLV-1) is a known aetiological agent of adult T-cell leukemia/lymphoma (ATL) and HTLV-1-associated myelopathy (HAM)/tropical spastic paraparesis (TSP) [2]; however, HTLV-2 is not clearly associated with a known clinical disease [1].

Though it has a worldwide distribution, HTLV-1 is endemic only in South Western Japan, the Caribbean Basin, West and Central Africa, some areas of intertropical Africa (such as South Gabon) and of the middle East (such as the Mashhad region in Northeastern Iran), rare isolated clusters in Australo-Melanesia, and foci in South America [3]. HTLV-2 is much less prevalent and has been predominantly reported among various American Indian populations like the Amerindians from Amazonia [4] and intravenous drug users from United States of America (USA) and Europe [5].

Serosurveys worldwide have documented that HTLV-1 and HTLV-2 are transmitted by contaminated blood transfusion [6], breast feeding [7], sharing of needles by intravenous drug users (IVDUs) [8], and unprotected sexual intercourse [9].

HTLV-1 and HTLV-2 virus transmission through blood transfusion is considered the most efficient route and the seroconversion of the recipients is around two months [10].

Seroconversion has been observed in 44%–63% of cases after receiving blood contaminated with HTLV-1 infected cells [11]. Efforts to disrupt its transmission have been taken in several countries, including the screening of blood donors for the presence of anti-HTLV-1/2 antibodies [12]. Screening of blood donors for HTLV-1/2 infection in Japan was implemented in 1986 [13], in the United States in 1988 [14], and in Continental France and Brazil in 1991 [15] and in 1993 [16], respectively. In 1995, Sweden decided to screen only the first time blood donors for anti-HTLV-1 due to the almost nonexistent local transmission of the virus [17]. Such screening is still under debate in other countries and is currently not being done in Uganda and in most other developing countries.

Screening has been shown to reduce risk of transfusion related transmission in USA [18]. It was in view of this that this study was undertaken. The aim of this study was to determine the prevalence of anti-HTLV-1/2 antibodies among the blood donors at Mbarara Regional Blood Bank, South Western Uganda.

## 2. Material and Methods

### 2.1. Study Site and Participants

The study was carried out at Mbarara Regional Blood Bank in Mbarara Municipality, South Western Uganda, from June 2014 to September 2014.

A total of 368 blood samples from the blood donors selected by consecutive nonprobability sampling method were included in this study. Blood donors were selected by the regional blood bank if they fulfilled all the criteria to be eligible for donation as described by the standard operating procedure of Uganda blood transfusion service. The participants' ages ranged between 15 and 51 years, and the median age was 18 years. The majority of the participants (>70%) were below 20 years of age because this regional blood bank repeatedly targets secondary school students to donate blood, most of whom happen to be below twenty (20) years of age. None of the blood donors were on immunosuppressive drugs or had a history of organ transplant. All blood samples from the blood donors enrolled in this study were tested for Hepatitis B surface antigen (HBsAg), anti-Hepatitis C, anti-Human Immunodeficiency Virus types 1 and 2 antibodies, and syphilis prior to HTLV-1/2 screening. The data extraction form was used to collect information regarding sociodemography, history of previous blood transfusion, blood donation, and level of education of the participant.

Sociodemography information including sex, age in years, occupation, place of birth, and the participant's (blood donor) place of residence location were recorded. Study participants were identified by the study number and no names were included.

### 2.2. Sample Size Determination

The sample size was determined using the Kish [19] formula of 1965 to give a 95% confidence interval:

(1) $$\begin{array}{c} n=\frac{{Z_{\alpha }}^{2}P\left(1-P\right)}{\delta^{2}}, \end{array}$$

where *n* is minimum sample size required; *Z*_*α*_ is standard normal deviate at 95% confidence interval corresponding to 1.96; *P* is assumed true population prevalence of HTLV-1/2 infections among blood donors at Mbarara Regional Blood Bank, South Western Uganda.

Since true population prevalence of HTLV-1/2 infections among blood donors at Mbarara Regional Blood Bank was not known, a HTLV seroprevalence of 2.3% obtained from a similar study done among blood donors in Mozambique (the country nearest to Uganda where a similar study had been done) by Caterino-de-Araujo et al., 2010, was used as the estimated prevalence; 1 − *P* is the probability of not having HTLV; *δ* is absolute error between the estimated and true population prevalence, Margin of Error (5%).

#### 2.2.1. Minimum Sample Size for Blood Donors

One has

(2) $$\begin{array}{c} n=\frac{{\left(1.96\right)}^{2}\times 0.977\times 0.023}{{\left(0.05\right)}^{2}}=35. \end{array}$$

From this formula the minimum number of blood samples required for screening to give this study sufficient power was 35.

However, a total of 368 blood samples were screened. A decision to include 368 samples was made because up to this number (368 samples) could be analysed in a single run with the ELISA kit that was used.

### 2.3. Inclusion and Exclusion Criteria for Blood Donors

#### 2.3.1. Inclusion Criteria

All donated blood was screened for Hepatitis B surface antigen, anti-Hepatitis C, anti-Human Immunodeficiency Virus types 1 and 2 antibodies, and syphilis at Mbarara Regional Blood Bank.

#### 2.3.2. Exclusion Criteria

All blood was submitted after the 368th sample.

### 2.4. Laboratory Testing

An enzyme-linked immunoassay was carried out for the determination of antibodies to HTLV-1 and HTLV-2 in serum and plasma using Murex HTLV-I + II DiaSorin, Dartford, UK (specificity of 99.5%), according to the manufacturer's recommendations. Screening of the blood samples for Hepatitis B surface antigen, anti-HCV antibodies, anti-HIV types 1 and 2 antibodies, and syphilis was done prior to anti-HTLV-1/2 testing. Initially reactive sera were confirmed by repeat testing and were considered positive only if they had double reactivity as the specificity of this test was high enough to confirm the infection.

### 2.5. Data Management

Data was double-entered in Microsoft Excel 2007 and was exported to Stata (Intercooled Stata 11, Stata Corporation, College Station, TX, USA) for analysis.

Univariate and bivariate analyses were done where appropriate. Descriptive statistical procedures were used to compute Chi-square for contingency tables.

The statistical inferences for the relationship between seropositivity and other factors were determined based on the Pearson Chi-square test of proportions' *p* value. The statistical significance was considered when the *p* value was below or equal to 0.05. Logistic regression was applied to estimate the magnitude of associations with risk factors for HTLV-1/2 seropositivity of the blood donors.

## 3. Results

### 3.1. Characteristics of Study Participants

A total of three hundred and sixty-eight blood donors participated in this study: 229 (62.2%) were males and 139 (37.8%) were females (Table 1). The donor age ranged from 15 to 51 years (median 18 and mode 17 years) and the biggest numbers of these were residing in a rural community, 287 (77.9%), whereas a few were residing in an urban community, 81 (22.1%). The majority were high school students (364 (98.9%)) while among others there were a businessman (0.3%), a driver (0.3%), a teacher (0.3%), and a housewife (0.3%) who had also attained education up to the secondary level with the exception of the housewife that had acquired education only up to primary level.

Of these 206 (55.98%) were first time donors, while 162 (44.02%) were repeat donors (Table 1).

The majority of the study participants were males who had attained a secondary level education.

**Figure 1:**
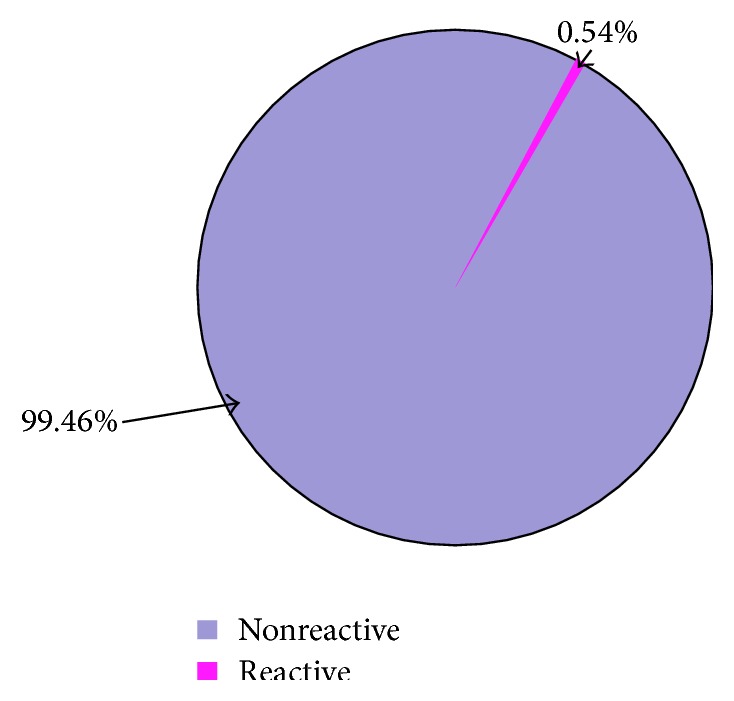
A pie chart showing HTLV-1/2 seropositivity of the study population.

Of the 368 blood donors, two (2) were found to be seropositive for HTLV-1/2 antibodies giving a prevalence of 0.54% (Figure 1). None of the female donors was found to be seropositive for HTLV-1/2.

The distribution of HTLV-1/2 seropositivity by community location or place of residence (rural versus urban), education, and occupation showed that the two HTLV-1/2 seropositive blood donors were both from rural areas (Table 2), had attained education up to secondary level (Table 3), and were students, respectively. Students recorded the highest participation (98.91%) in this study as well. However, the relationship between HTLV-1/2 seropositivity and occupation was statistically not significant (*χ* ^2^  (4 df) = 0.0221, *p* = 1.0).

All variables from the univariate analysis including age, sex, place of birth, community location of place of residence (rural versus urban), occupation, and level of education were entered into multiple logistic regression models. In this analysis, no independent risk factors were found to be significantly associated with HTLV-1/2 seropositivity (*χ* ^2^  (4 df) = 0.34, *p* = 0.99).

## 4. Discussion

The seroprevalence of HTLV-1/2 in this study (0.5%) is similar to that found among blood donors presenting to blood banks in Ghana [20] where blood donors were also screened using an enzyme immunoassay. But this seroprevalence differs from studies among blood donors in Mozambique and Nigeria where the seroprevalences of HTLV-1/2 were found to be 0.9% [21] and 3.6% [22], respectively. In both studies, that is, one carried out in Mozambique and the other in Nigeria, serum samples were assessed for HTLV-1/2 specific antibodies by using enzyme immunoassays, and infections with HTLV-1 and HTLV-2 were confirmed by using Western blot.

The seroprevalence in this present study is comparatively low. Probably, this could be due to the fact that the majority of the participants in this study were secondary school students who are at a low risk for HTLV-1/2 infection while in the other studies [21, 22] there was a wide variation in occupations of the study participants.

In this study, all HTLV-1/2 seropositive donors were males. This is not in conformity with studies that have shown prevalence of HTLV-1 and HTLV-2 to be higher in females as opposed to males which is attributed to more efficient transmission from men to women during sexual intercourse [23].

This is most likely due to the higher proportion of males compared with females in this study. This is a limitation of the study as gender of the participants was skewed in favour of the male sex.

The two HTLV-1/2 seropositive blood donors were both from rural areas which were their places of origin as well. This finding is similar to that observed in studies done in Gabon and Mozambique by Le Hesran et al. [24] and Caterino-de-Araujo et al. [25], respectively. HTLV-1/2 seropositivity among the rural and not urban residents could suggest an ancient presence of HTLV in the rural areas where these seropositive donors come or specific lifestyle factors in a rural environment that puts one at risk for acquisition of HTLV.

Having a history of crusted scabies caused by massive infestation with* Sarcoptes scabiei* var.* Hominis* as suggested by other studies has also been found to be associated with HTLV seropositivity among rural communities [26, 27].

In this study it was not possible to further explore specific lifestyle factors associated with risk of acquiring HTLV infection in this group (rural dwellers) due to the small proportion of seropositive donors from the rural area.

In this study, all HTLV-seropositive donors had attained education up to secondary level. Generally, the distribution of educational attainment was quite narrow and was almost identical between the seropositive and seronegative donors, perhaps reflecting a disadvantage of studying a more or less occupational based cohort. Thus, the study was unable to confirm any associations between HTLV-I/2 seropositivity and education, a marker for socioeconomic status (*p* = 0.941), despite suggestions of such relations in previous studies [25, 28].

Some occupations have been pinpointed to be associated with high prevalence of HTLV-1/2 infections [22]. In this study, all HTLV-1/2 seropositive participants (2, 0.6%) were students. This occupation (high school students) has not been associated with coming in contact with blood or blood products and review of the literature reveals no other reports of such an association between HTLV-1/2 seropositivity and being a student at this level. A possible explanation could be that these seropositive donors may have had sex with a partner that could have been infected with HTLV. There could be other factors contributing to this finding but they were not looked for in this study.

Also, none of the 2 seropositive blood donors had been previously transfused; no association as such can be established between transfusion history and HTLV-1/2 positivity among these blood donors as well.

## 5. Limitations

This study has a number of limitations. First, the number of the studied blood donors was low (368); therefore, the seroprevalence may have been underestimated and thus the result may not really be informative on an epidemiological and public heath level. Second, a higher proportion of males (62.2%) compared with females (37.8%) participated in this study. Since it is known that HTLV-1/-2 seroprevalence is higher in women than in men; the higher proportion of males in this study may also have led to an underestimation of the prevalence of HTLV-1/2 in females as opposed to males.

Third, most samples were obtained from individuals less than 20 years old; this very likely led to an underestimation of the real HTLV-1/2 prevalence since it is known that HTLV-1/-2 seroprevalence is higher in older individuals than in younger ones. Finally, the reported HTLV-1/-2 prevalence was obtained using only a commercial EIA (specificity > 99.5%), and a confirmatory test (Western blot) or PCR was not done to confirm the results.

## 6. Conclusion

Prevalence of HTLV 1/2 among blood donors at Mbarara Regional Blood Bank is too low to provide justification for screening of blood donations.

## Figures and Tables

**Table 1:** Demographic and donor baseline characteristics for the study participants.

Characteristic	*N*	(%)
Sex		
Male	229	62.2
Female	139	37.8
Age, years		
** <**20	273	74.2
** **20–40	93	25.3
** **>40	2	0.5
Education		
** **Primary	1	0.3
** **Secondary	367	99.7
** **Tertiary	0	0
Occupation		
Student	364	98.9
Teacher	1	0.3
Business	1	0.3
Driver	1	0.3
Other (housewife)	1	0.3
Marital status		
Single	364	98.9
Married	4	1.2
Address of residence		
Rural	287	77.9
Urban	81	21.1
Type of donor		
New	206	55.98
Repeat	162	44.02

**Table 2:** HTLV-1/2 seropositivity by rural versus urban location.

Community location	Number of participants tested (%)	Number of samples reactive for HTLV-1/2 antibodies	Total (%) HTLV-1/2 seropositivity
Rural	287 (77.99)	2	2 (0.7)
Urban	81 (22.01)	0	0 (0)
Total	368 (100)	2	2 (0.54)

**Table 3:** HTLV-1/2 seropositivity by the level of education.

Level of education	Number of participants tested	Number of samples reactive for HTLV 1/2 antibodies	Total (%) HTLV-1/2 seropositivity
Primary	1	0	0 (0)
Secondary	367	2	2 (0.54)
Tertiary	0	0	0 (0)
Total	368	2	2 (0.54)
